# Changing hands: persistent alterations to body image following brief exposure to multisensory distortions

**DOI:** 10.1007/s00221-017-4935-2

**Published:** 2017-03-14

**Authors:** A. Treshi-marie Perera, Roger Newport, Kirsten J. McKenzie

**Affiliations:** 1 School of Psychology, University of Nottingham Malaysia Campus, Jalan Broga, Semenyih, Selangor Darul Ehsan 43500 Malaysia; 2 School of Psychology, University of Nottingham UK Campus, University Park, Nottingham, NG7 2RD UK; 3 School of Psychology, University of Lincoln, Brayford Pool, Lincoln, LN6 7TS UK

**Keywords:** Body representation, Multisensory illusions, MIRAGE, Body ownership, Embodiment, Finger stretching

## Abstract

The dynamic flexibility of body representation has been highlighted through numerous lines of research that range from clinical studies reporting disorders of body ownership, to experimentally induced somatic illusions that have provided evidence for the embodiment of manipulated representations and even fake limbs. While most studies have reported that enlargement of body parts alters somatic perception, and that these can be more readily embodied, shrunken body parts have not been found to consistently alter somatic experiences, perhaps due to reduced feelings of ownership over smaller body parts. Over two experiments, we aimed to investigate the mechanisms responsible for altered somatic representations following exposure to both enlarged and shrunken body parts. Participants were given the impression that their hand and index finger were either longer or shorter than veridical length and asked to judge veridical finger length using online and offline size estimation tasks, as well as to report the degree of ownership towards the distorted finger and hand representations. Ownership was claimed over all distorted representations of the hand and finger and no differences were seen across ownership ratings, while the online and offline measurements of perceived size demonstrated differing response patterns. These findings suggest that ownership towards manipulated body representations is more bidirectional than previously thought and also suggest differences in perceived body representation with respect to the method of measurement suggesting that online and offline tasks may tap into different aspects of body representation.

## Introduction

Understanding the mechanisms by which internal somatic representations are formed and maintained has important implications for understanding our physical interactions with the external environment. Despite the subjective stability of somatic representations, numerous misinterpretations of somatic experiences have been reported following damage to cortical regions including the premotor and parietal regions that are associated with maintaining an accurate body representation (Tsakiris 2010). For instance, clinical studies of asomatognosia have shown that patients with acquired brain injury report disownership of their body or body parts (Arzy et al. 2006a) which in some cases can also be attributed to another individual (somatoparaphrenia; Bisiach et al. 1991; Vallar and Ronchi 2008).

Although such somatic distortions may appear to be features of pathological conditions, recent research has demonstrated that distorted somatic experiences are indeed characteristic of healthy body representations as well. Large distortions in body size are often reported in *body image* tasks that require participants to compare the perceived size of body parts (e.g. the hand, finger) to the length of a line or in tasks requiring participants to localise in external space different landmarks (e.g. fingertip) of their occluded hand (Longo and Haggard 2010, 2012). Additional evidence for distorted somatic experiences has also been demonstrated following experimental manipulation of perceived shape and size of the body. For example, vibration of the biceps and triceps tendons has been found to give rise to an illusory extension and flexion of the forearm, respectively, creating a feeling that the limb has been moved or displaced (Lackner 1988). In addition, following synchronous visuo-tactile stimulation, many virtual reality studies have provided evidence for ownership towards both larger and smaller bodies (van der Hoort et al. 2011; Banakou et al. 2013). Collectively, these findings suggest that healthy body representations can be readily updated, based on incoming sensory information that gives rise to altered somatic experiences.

Studies investigating the flexible and modifiable nature of internal bodily representations have reported large distortions in somatic perception following manipulations to perceived body shape and size. For example, Bruno and Bertamini (2010) found that manipulating perceived hand size altered the perceived size of held objects, such that objects were judged to be smaller or larger following exposure to enlarged and reduced models of the hand, respectively. Using head-mounted displays, van der Hoort et al. (2011) demonstrated that owning a smaller body resulted in objects being perceived to be larger, whereas the opposite effect was seen when participants felt ownership over a larger body. Perception of the external environment (e.g. visual perception of objects and distances) may, therefore, depend on one’s perceived body representation, which provides a sense of scale. Similar scaling effects are also reported in virtual environments following the embodiment of different sized hands and bodies. In a series of experiments, Linkenauger et al. (2013) demonstrated that the hand is used as a metric to scale the size of surrounding objects and that modifying the dimensions of the hand’s representation altered the perceived size of objects. Using immersive virtual reality, Banakou et al. (2013) found that embodiment of a virtual toddler body led to significantly greater overestimations of object size as compared to embodiment of a scaled down adult body. However, it should be noted that these effects of altered somatic perception following manipulations to body shape and size have been found to be rather inconsistent. For instance, Haggard and Jundi (2009) found weight of a grasped object to be influenced by perceived hand size only following exposure to enlarged representations of the hand. In line with this finding, de Vignemont et al. (2005) found reduced tactile two-point discrimination thresholds following illusory elongation of perceived finger size, whereas no difference was seen following illusory shrinking. Moreover, in a modified version of the rubber hand illusion (RHI; Botvinick and Cohen 1998) that involved video footage of the real hand, Pavani and Zampini (2007) only found the illusion to be elicited following exposure to veridical and enlarged representations of the hand. These findings, therefore, demonstrate asymmetric tendencies to acknowledge and integrate enlarged (or veridical) body parts into our body representation, thus creating a need to more closely inspect and further understand the mechanisms underlying the varied effects of such somatic illusions. The failure to produce alterations in somatic experiences with shrunken body parts in previous studies may suggest a lack of ownership over smaller body parts (Ramachandran and Rogers-Ramachandran 2007) perhaps due to bodily changes in the form of elongation or extension being more frequent and rapid (Pavani and Zampini 2007; Haggard and Jundi 2009). Moreover, a majority of these previous studies have been limited to depictions of body parts that did not allow dynamic changes in perceived body size and were, therefore, less realistic in appearance. As a result, given our reduced familiarity with shrunken body parts, such representations would have been less likely to be incorporated into the body representation.

In the current studies, the MIRAGE-mediated reality system (Newport et al. 2009; University of Nottingham) was used to create spatially coincident *dynamic* (i.e. moving) multisensory illusions in which real-time visual, tactile and proprioceptive sensory information altered perceived hand and index finger size in both directions, creating stretched and shrunken representations of the participant’s own hand and finger. The incorporation of dynamic multisensory illusions in this case implies that the viewed images are under participants’ control during active movement, perhaps, enabling even shrunken depictions of a body part to be more readily incorporated into the body representation (Newport et al. 2010). Such dynamic representations also provide greater ecological validity compared to traditional experimental setups which included static stimuli, inducing greater feelings of ownership and/or agency over the limb than less realistic representations and may, therefore, be more easily incorporated into the body representation. Indeed, emotion recognition literature has suggested that emotions are better recognised and rated to be more realistic and intense with dynamic stimuli compared to static stimuli in both healthy and patient populations (Harwood et al. 1999; Weyers et al. 2006). Additionally, studies have also revealed greater activity in the visual and temporal cortices following exposure to dynamic compared to static stimuli (Kilts et al. 2003) perhaps due to greater availability of information in dynamic displays.

The main aim of the current experiments was to, therefore, explore the mechanisms responsible for distorted somatic perception following exposure to dynamic multisensory illusions of perceived own body size. In both Experiments 1 and 2, we examined whether participants’ perception of their veridical body size was altered following exposure to such size-altering illusions via subjective ratings of illusion strength and ownership using standard questionnaire methods. As a secondary aim, the latter experiment also examined whether any of the observed effects of updated body size were affected by the method by which body size was assessed.

## Experiment 1

In this study, participants were instructed to judge their veridical (or real) hand and finger size following exposure to illusory stretched/shrunken representations of their hand and finger to examine whether judgements of own body perception were influenced by the nature of the illusions. If body representation is influenced by the illusions, perceived veridical body size would be expected to update in the direction of the illusory manipulation, with longer and shorter representations of the finger and hand judged as normal size following illusory stretching and shrinking, respectively. Illusion strength and ownership were measured using questionnaires that assessed how strongly participants felt each illusion and how strongly they felt the distorted representations of their finger and hand to belong to them, respectively. Given the dynamic nature of the illusions employed, participants were expected to experience each illusion strongly and claim ownership over these manipulated representations of their hand.

### Experiment 1: Method

#### Participants

Thirty-seven right-handed (Oldfield 1971) participants (17 male) aged 18–29 years (mean age = 21.89; SD = 2.67) were recruited. Written informed consent was obtained prior to participation and none of the participants reported any sensory deficits. All procedures were approved by the University of Nottingham Malaysia Campus Research Ethics Committee and carried out in accordance with the Declaration of Helsinki.

#### Apparatus and material

##### MIRAGE system

**Fig. 1:**
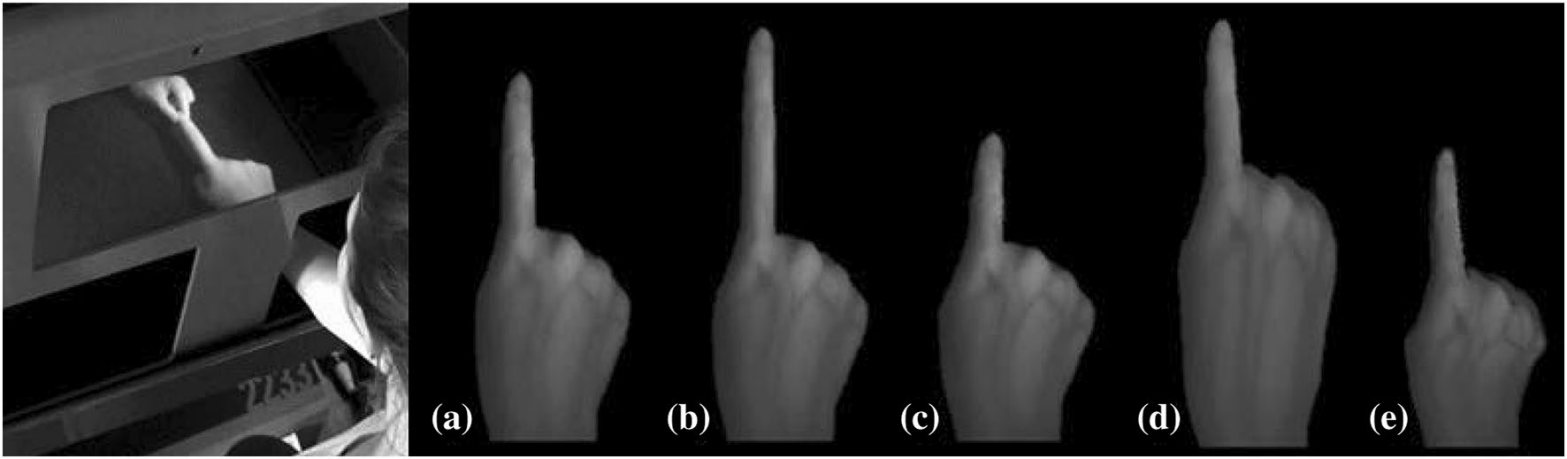
Multisensory illusions: **a** veridical condition (no manipulation), **b** stretched finger, **c** shrunken finger, **d** stretched hand, **e** shrunken hand

The MIRAGE system uses mirrors and cameras to provide participants with real-time video footage of their own hand in its actual location (Newport et al. 2010) with a delay less than 17 ms (Newport et al. 2009, 2010). In the current study, the captured images were manipulated by custom software (Preston and Newport 2011) to create four illusions: stretched finger, shrunken finger, stretched hand and shrunken hand (see Fig. 1). During the stretched illusions, the experimenter grasped and pulled the participant’s index finger/hand with slight pressure, while the image of their finger/hand (seen through the device) was simultaneously seen to grow longer. Perceived finger length and hand length were both stretched by approximately 50 mm. For the shrunken illusions, participant’s finger/hand was gently pushed in with light pressure while the image of the hand/finger was simultaneously seen to grow shorter (Preston and Newport 2011). Again, hand length was reduced by approximately 50 mm, while finger length was shrunken by approximately 37 mm. When the finger was being stretched and shrunken the distal end of the index finger (fingertip) was grasped and pulled/pushed, whereas when the hand was stretched and shrunken the dorsal region of the palm was grasped and pulled/pushed.

#### Questionnaire measures

Participants responded to two questionnaire measures. (1) The *acclimatisation questionnaire* (Newport et al. 2010) was a six-item questionnaire used to confirm whether or not participants had a strong feeling of ownership towards the video image of their hand when seen through the MIRAGE-mediated system prior to the illusions (example questionnaire items: ‘It seemed like the image of the hand was my own’, ‘It seemed like the image of the hand belonged to me’). (2) *Illusion strength and ownership questionnaires* assessed the extent to which each illusion was incorporated into participants’ body representation (example questionnaire items: ‘I felt like my finger/hand was really being stretched/shrunken’) as well as participants’ sense of ownership towards the distorted appearance of their finger and hand (‘I feel like I am watching myself’; Preston and Newport 2012). In all questionnaires, participants made verbal judgements on a 9-point numeric rating scale in which 9 indicated strong illusion strength/ ownership and 1 indicated low illusion strength/ ownership.

#### Procedure

Upon being seated in front of the MIRAGE system, participants were given a brief period of acclimatisation (~20 s) during which time they were encouraged to move both hands within the device in any way they wanted (no systematic instructions were given). This was followed by the 6 item acclimatisation questionnaire. Participants were then instructed to take their left hand out and the first illusion (stretched finger, shrunken finger, stretched hand, or shrunken hand) was conducted in a counter-balanced order on the right hand. As mentioned above, during each illusion, the experimenter either gently pulled or pushed participants’ finger/hand while they watched their finger/hand grow longer or shorter than its veridical length. Each illusion took approximately 5 s to administer and participants were instructed to keep their hands still during and following each multisensory illusion. After the application of each illusion, the experimenter reached for each participant’s finger/hand and asked them whether they felt the touch, with the aim of providing congruent visuo-tactile feedback to indicate that participants were still watching their own hand. Illusion strength and ownership questionnaires corresponding to each multisensory illusion condition were then conducted and took approximately 45 s. Participants’ judgements of perceived veridical finger and hand length were then obtained; participants were asked whether these stretched and shrunken representations (following illusory stretching and shrinking, respectively) of the finger/hand had to be made longer or shorter to reach its veridical length. The experimenter then grasped and manually pulled/pushed participants’ finger/hand in the direction specified, while the visual representation of the hand/finger was simultaneously increased/decreased in size one unit at a time (units are defined in terms of screen pixels, for which 1 pixel = 1.5 mm), in a step-wise manner that had the visual appearance of slow but continuous growth or shrinking. Participants were instructed to say ‘Stop!’ when they felt like their finger/hand had reached its ‘normal’ veridical length (“say ‘Stop!’ when you feel like your finger/hand reaches its real length”). At this verbal response, the experimenter immediately released the participant’s finger/hand and ceased to apply any further visual manipulations. This stopping point was recorded and used to calculate the percentage increase or decrease in perceived finger length following the illusion, compared to initial finger length. Participants were asked to take their hand out of the MIRAGE system at the end of every illusion condition and allowed to move it in order to reset perceived finger length and prevent any carryover effects from the previous illusion.

### Experiment 1: Results

#### Questionnaire responses

Acclimatisation and illusion strength questionnaire ratings were not normally distributed (Shapiro Wilk statistic; *p* < 0.05) and remained so despite attempts to transform the data; consequently, non-parametric analyses were conducted.

##### Acclimatisation questionnaire

**Fig. 2:**
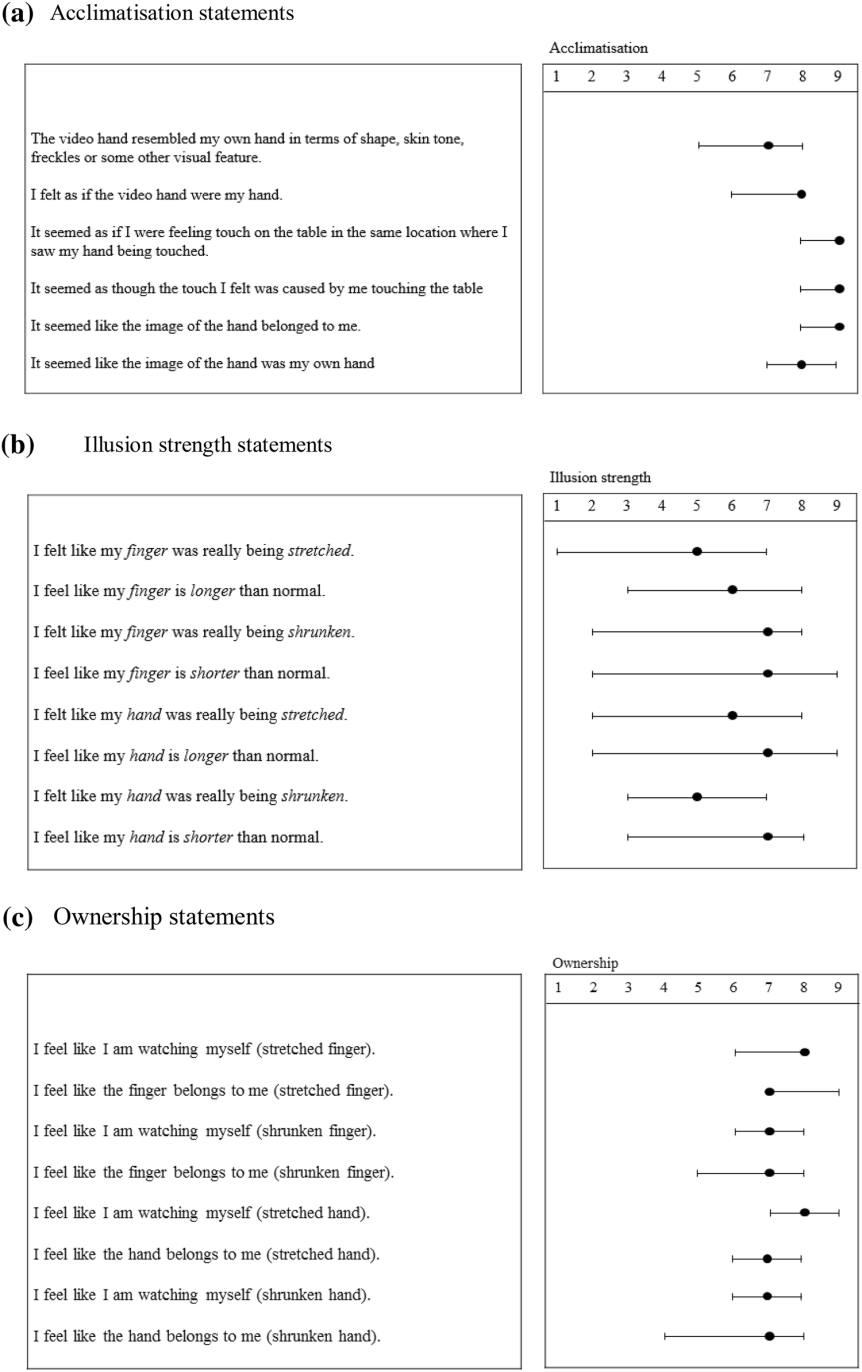
Medians and inter-quartile ranges for questionnaire ratings: **a** acclimatisation ratings, **b** illusion strength ratings, **c** ownership ratings

Mean ratings for statements on the acclimatisation questionnaire indicated a strong sense of ownership towards the live video images of the hands (see Fig. 2a). Participants strongly agreed with statements such as ‘It seemed like the image of the hand was my own’ (median = 9) and ‘It seemed like the image of the hand belonged to me’ (median = 8).

##### Illusion strength and hand ownership questionnaires

Mean ratings for each statement indicated that participants felt their finger to be stretched or shrunk but still claimed ownership over these manipulated representations of the finger (see Fig. 2b, c). Responses to the statements *‘*I felt like my finger/hand was being stretched/shrunken’ and ‘I feel like my finger/hand is longer/shorter than normal’ were separately averaged for the stretched and shrunken finger/hand conditions to obtain mean ratings for illusion strength—that is, the extent to which participants felt each multisensory illusion. Similarly, ratings to the statements ‘I feel like I am watching myself’ and ‘I feel like the finger/hand I am seeing belongs to me’ were separately averaged for each illusion condition to obtain mean ratings of ownership over the manipulated representations of the finger and hand. Mean ownership ratings indicated that 95 and 89% of participants had ratings of 5 or higher during the stretched and shrunken finger conditions, respectively. For illusion strength, 68% of participants had average ratings of 5 or above during the stretched finger condition, while 70% of participants had ratings of 5 and above during the shrunken finger condition.

During the stretched hand condition, 92% had mean ownership scores of 5 or greater while 86% had mean ownership scores of 5 and above during the shrunken hand illusion condition. Mean illusion strength ratings indicated that 70% of participants had ratings of 5 and above while 73% had mean ratings of 5 and above during the shrunken hand condition. Less than 30% of the sample had scores of the 3 or less in all conditions, demonstrating that a majority of the sample strongly felt each illusion and claimed strong ownership over their hand and finger regardless of the direction of the distortion.

Mean illusion strength and ownership ratings were then compared across the four conditions. A Friedman’s ANOVA revealed no significant differences for mean illusion strength (*χ* ^2^ (3, *N* = 37) = 4.00, *p* = 0.26) or mean ownership (*χ* ^2^ (3, *N* = 37) = 3.18, *p* = 0.36) across the four conditions.

#### Judgments of perceived finger length: online resizing

**Fig. 3:**
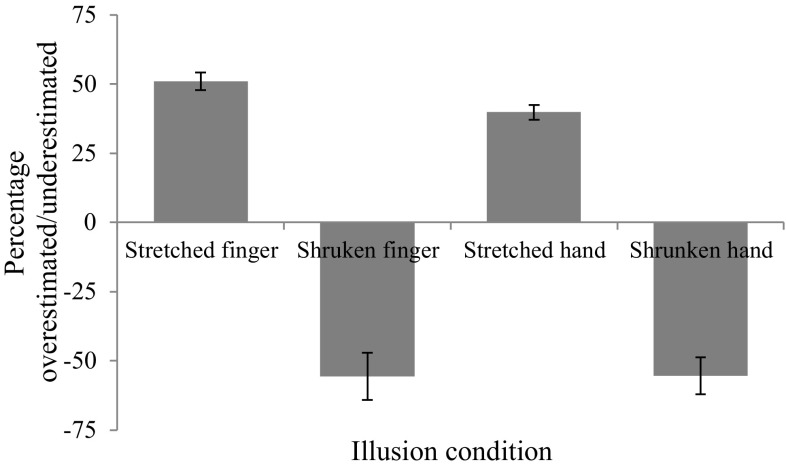
Percentage overestimation/underestimation of finger and hand length following illusory manipulations (*error bars* show standard error of the mean)

Percentage increase or decrease in finger length from veridical was calculated and used to determine the mean percentage overestimation/underestimation (from veridical size) for each participant in all four conditions (see Fig. 3). Overestimation and underestimation scores remained not normally distributed (Shapiro Wilk statistic; *p* < 0.05); hence, non-parametric analyses were conducted. A Friedman’s ANOVA revealed no significant differences across percentage overestimation and underestimation across the four illusion conditions (*χ* ^2^ (3, *N* = 37) = 5.47, *p* = 0.14).

Chi square analyses were then used to compare the proportion of participants that overestimated or underestimated perceived finger and hand length in each condition to those that did not. Following the stretched finger illusion, all participants (100%) overestimated perceived finger length (mean percentage overestimation = 50.99%, SD = 19.87) stating that their finger had reached its veridical length when it was still much longer than in reality. 68% of participants (*χ* ^2^ (1, *N* = 37) = 4.57, *p* = 0.033) underestimated their finger length following the shrunken finger illusion (mean percentage underestimation = 55.74%, SD = 51.97). All participants (100%) also overestimated perceived hand length following the stretched hand illusion (mean percentage overestimation = 39.86%, SD = 16.27), while 73% of participants (*χ* ^2^ (1, *N* = 37) = 7.81, *p* = 0.005) underestimated the perceived length of their hand following the shrunken hand illusion (mean percentage underestimation = 55.40%, SD = 40.39). One sample *t* tests revealed that perceived length overestimated during the stretched finger (*t*_(36)_ = 15.06, *p* < 0.001, *d* = 2.47) and stretched hand (*t*_(36)_ = 14.86, *p* < 0.001, *d* = 2.44) conditions, as well as the perceived length underestimated during the shrunken finger (*t*_(24)_ = 8.14, *p* < 0.001, *d* = 1.63) and shrunken hand conditions (*t*_(26)_ = 8.81, *p* < 0.001, *d* = 1.69), were all significantly greater than zero (that is, significantly larger or smaller than veridical finger length).

Correlation analyses were finally conducted between mean illusion strength ratings and percentage overestimation/underestimation for all conditions; however, no significant association between illusion strength and judgements of perceived finger/hand length was observed for any of the conditions (all *p* > 0.05).

### Experiment 1: Discussion

This study investigated how illusory manipulations of body size altered perceived body representation and the underlying mechanisms. In line with our hypothesis, perceived veridical body size following each multisensory illusion was affected by the nature of that illusion with longer and shorter fingers and hands being judged as veridical length following illusory stretching and shrinking, respectively. The findings, therefore, suggest that each illusion may have temporarily altered the mental representations of the hand and finger. Furthermore, our results expand upon previous studies that have found shrunken/minified body parts to alter object perception in the external environment (Bruno and Bertamini 2010; Banakou et al. 2013), by demonstrating that a brief exposure to stretched and shrunken body parts also alters the perceived size of one’s own body—therefore, the flexibility of the body representation could perhaps be more bidirectional than previously thought (Pavani and Zampini 2007; deVignemont et al. 2005). The questionnaire data revealed no significant differences in ownership across the conditions indicating that ownership was not lost as a result of the multisensory distortions. In fact strong ownership was claimed over both shrunken illusions. Illusion strength ratings were not associated with judgements of veridical finger judgements; nevertheless, these findings complement and extend previous studies that have provided evidence for ownership towards different body forms (van der Hoort et al. 2011; Banakou et al. 2013) and provide evidence for the dynamic flexibility of the internal body representation.

Given that body representation was updated in the direction of the distortion, following illusory stretching and shrinking, one may argue that, rather than reflecting any influence of a ‘directional’ distortion of the body representation, perhaps there may have been an acceptable range, within which body-part sizes were considered to be veridical, or at least close enough to veridical to have been accepted as ‘normal’ following illusory stretching and shrinking. Therefore, the resultant change in body size may simply reflect a tendency for participants to say ‘stop’ at the higher or lower end of this range; displaying a bias toward saying “stop” early following each manipulation. This possibility was addressed in Experiment 2.

## Experiment 2

To explore any bias toward simply accepting a distorted body part as ‘close enough’ to normal, and demonstrate that the multisensory illusions were in fact responsible for changes in perceived finger length and hand size, the current study introduced a stepwise size manipulation following illusory stretching and shrinking. Additionally, given that previous studies have reported discrepancies in perceived body shape and size with regard to the methods of measurement (Cash and Deagle 1997; Longo and Haggard 2012), Experiment 2 also included an additional *offline measure* of perceived body size that assessed alterations to the body representations post-illusion. For example, perceived body representation is found to be different with depictive tasks (in which shape and size of the body is compared to visual depictions of that body part) and metric tasks (in which body size is compared to a non-body physical standard) in both healthy and clinical populations (Cash and Deagle 1997; Longo and Haggard 2012) suggesting that these measures may reflect different aspects of the body representation. Previous virtual reality studies appear to have adopted a form of an *online* measure when determining somatic perception in the virtual environment, as judgements of object size perception were made during the course of the manipulation which did not necessitate access to stored (offline) body representations (e.g. van der Hoort et al. 2011). Indeed, offline body representations are thought to be stable and reflect how the body is usually perceived to be (Carruthers 2008). Therefore, while findings in line with Experiment 1 were expected for the online measure, the offline measure was not expected to be influenced by the illusions. As no significant differences in overestimation and underestimation were observed across the illusory manipulations, Experiment 2 focused solely on illusory stretching and shrinking of the right index finger.

### Method

#### Participants

Twenty-three right handed (Oldfield 1971) participants (9 male) aged 18–21 years (mean age = 19.00; *SD* = 0.77) were recruited. Participants reported no sensory deficits and gave written informed consent prior to participation.

#### Apparatus and material

##### MIRAGE system

**Fig. 4:**
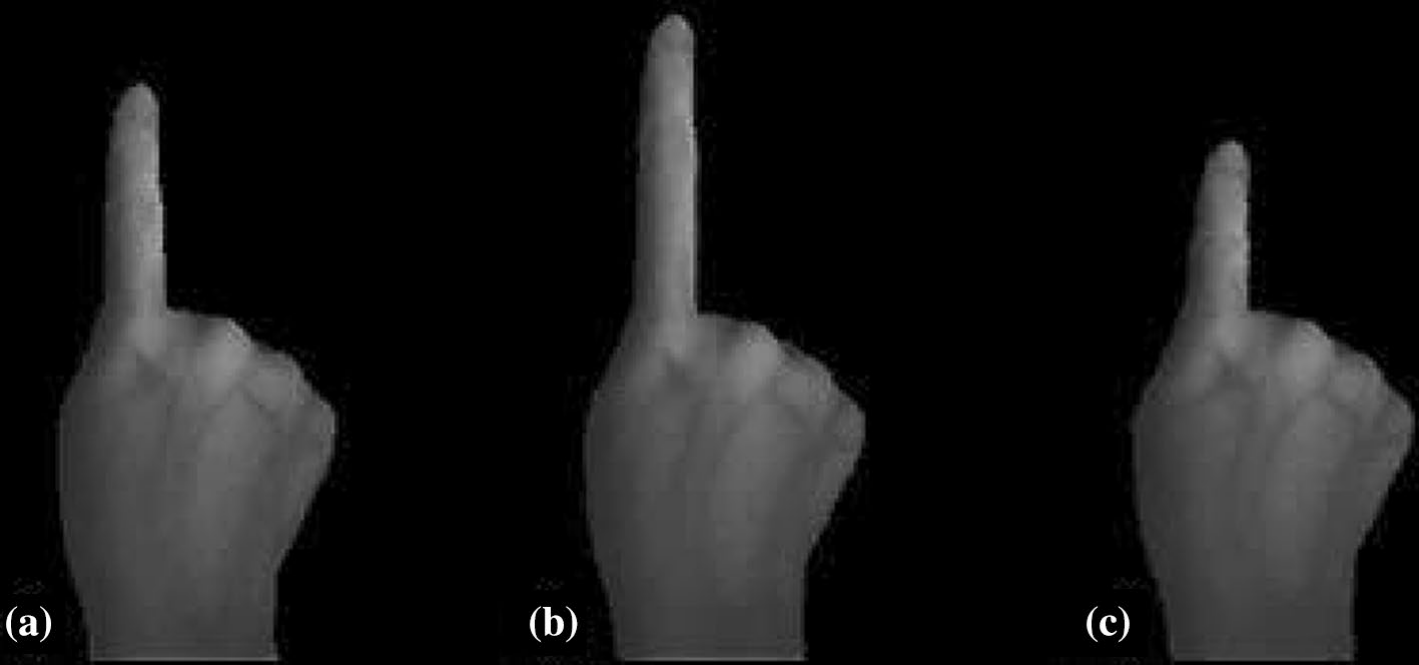
Veridical finger length and manipulated length (using multisensory illusions). **a** Veridical finger length, **b** stretched finger, **c** shrunken finger

As in Experiment 1, during the stretched and shrunken finger conditions the experimenter gently pulled or pushed participants’ index finger with light pressure while the image of the finger was seen to grow longer and shorter, respectively (see Fig. 4). Finger length was stretched by approximately 60 mm and shrunken by 50 mm.

#### Questionnaire measures

As in Experiment 1, the acclimatisation and illusion strength and hand ownership questionnaires were used to assess the extent to which participants felt ownership over a video image of their hand, as well as how strongly participants incorporated the manipulated representations of their hand into their body representation.

#### Procedure

**Fig. 5:**
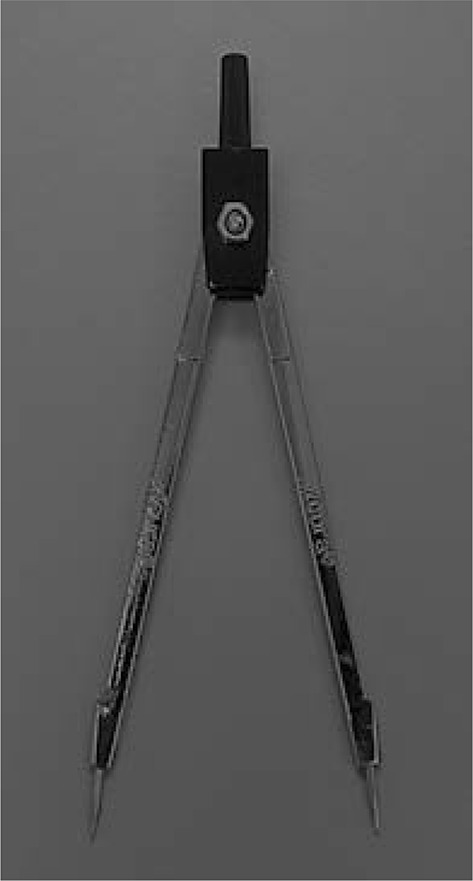
Mathematical divider—the Oxford Mathematical set of instruments; Helix-England

Following a brief period of acclimatisation during which time both hands were viewed to move freely within the MIRAGE system (~20 s) the acclimatisation questionnaire was administered. Participants were then asked to take their left hand out and handed a closed or opened (counterbalanced between participants) divider tool (Fig. 5) from a mathematical drawing kit (The Oxford Mathematical set of instruments; Helix-England). They were instructed to open or close the divider using the left hand, until the distance between the two points was felt to match the perceived length of the un-manipulated right index finger (*initial baseline length; accuracy 1 mm*). Although participants could see the hand that was placed within the MIRAGE system, they were encouraged to move the two points of the divider to demonstrate how long they *felt* their index finger to be (and not what they were seeing) to provide a baseline measurement.

**Fig. 6:**
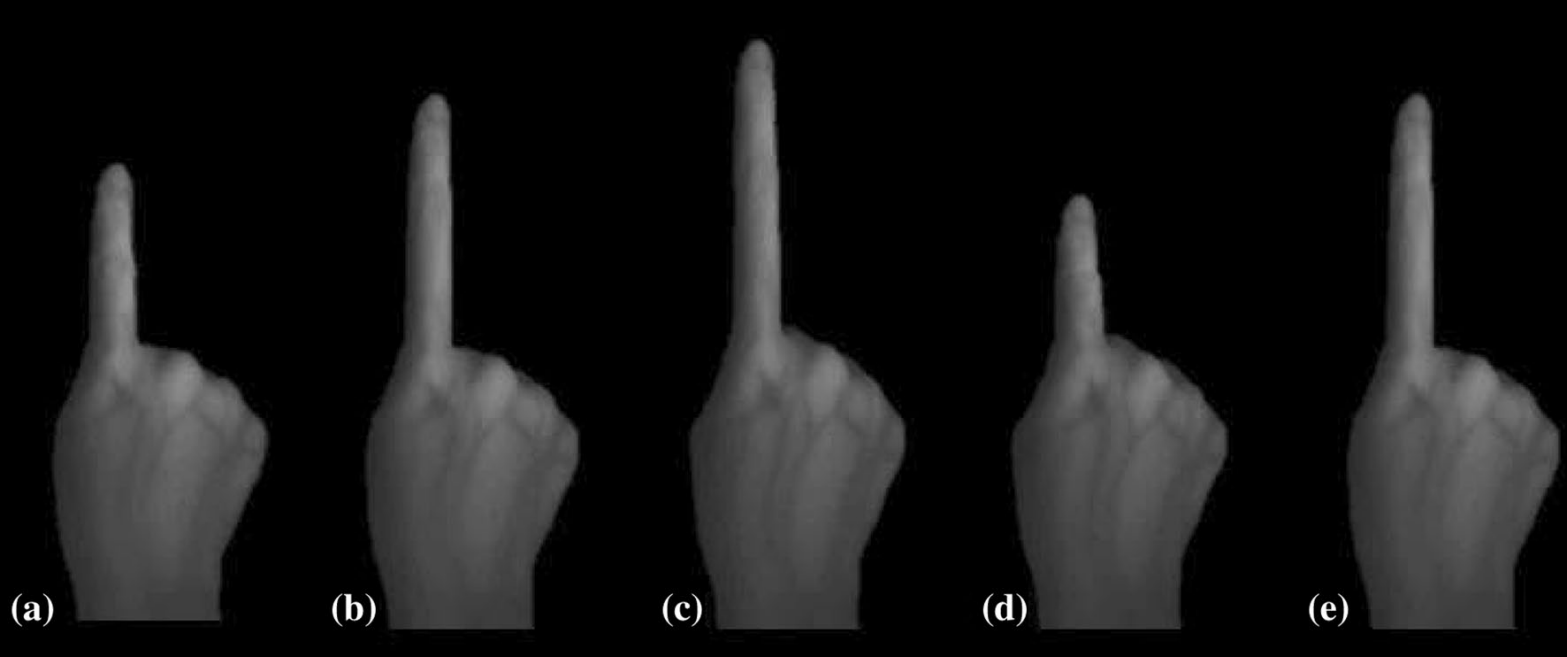
Example of stretch-shrink-stretch stepwise manipulation. **a** Veridical length, **b** initial stretched length, **c** stretched to half the number of units of the initial stretching, **d** shrunken to half the number of units of the initial stretched length, **e** stretched back to original stretched length

The first visuo-proprioceptive illusion (stretched finger/shrunken finger) was then conducted in a counter-balanced order on the right hand and was followed by corresponding illusion strength and hand ownership questionnaires. Illusion administration was identical to the procedure described in Experiment 1. Following the illusion strength and hand ownership questionnaires, the experimenter used a stepwise manipulation to change the (already manipulated) length of the index finger in the following sequence; stretch–shrink–stretch for half the trials and shrink–stretch–shrink in the remainder. During the stretch–shrink–stretch step-wise manipulation, the altered finger length (e.g. 30 units) was further stretched by half the number of units of the initial altered length [e.g. (30 + 15) 45 units], then shrunken by half the number of units of the initial length altered [e.g. (45 − 30) 15 units] and stretched again by half the number of units of the initially altered length which brought the finger back to initial manipulated length [e.g. (15 + 15) 30 units] and vice versa for the shrink–stretch–shrink manipulation (see Fig. 6 for example). The stretching and shrinking were similar to that described previously and took approximately 10 s. At each point during the stepwise manipulation, the experimenter reached and touched the tip of the finger ensuring congruency in what participants felt and saw. The stepwise manipulation was followed by veridical finger length judgements. As in Experiment 1, participants indicated whether their finger had to be made longer or shorter to reach its ‘normal’ veridical length, while the experimenter altered perceived finger length in the direction specified. The stopping point was recorded and used to calculate the percentage increase or decrease in perceived finger length following each illusion. To further examine the effectiveness of the illusion, all participants were again handed the divider tool^1^and asked to judge the size they felt the real length of their finger to be following each illusion, using the same procedure as the baseline, and thus providing an offline measure of perceived body size. Each illusion was repeated three times for each participant, and participants were asked to take their hand out of the MIRAGE system at the end of every trial to reset perceived finger length. As each illusion was repeated three times, illusion strength and ownership statements were presented in a randomised order in every trial.

### Results

#### Questionnaire responses

##### Acclimatisation questionnaire

**Fig. 7:**
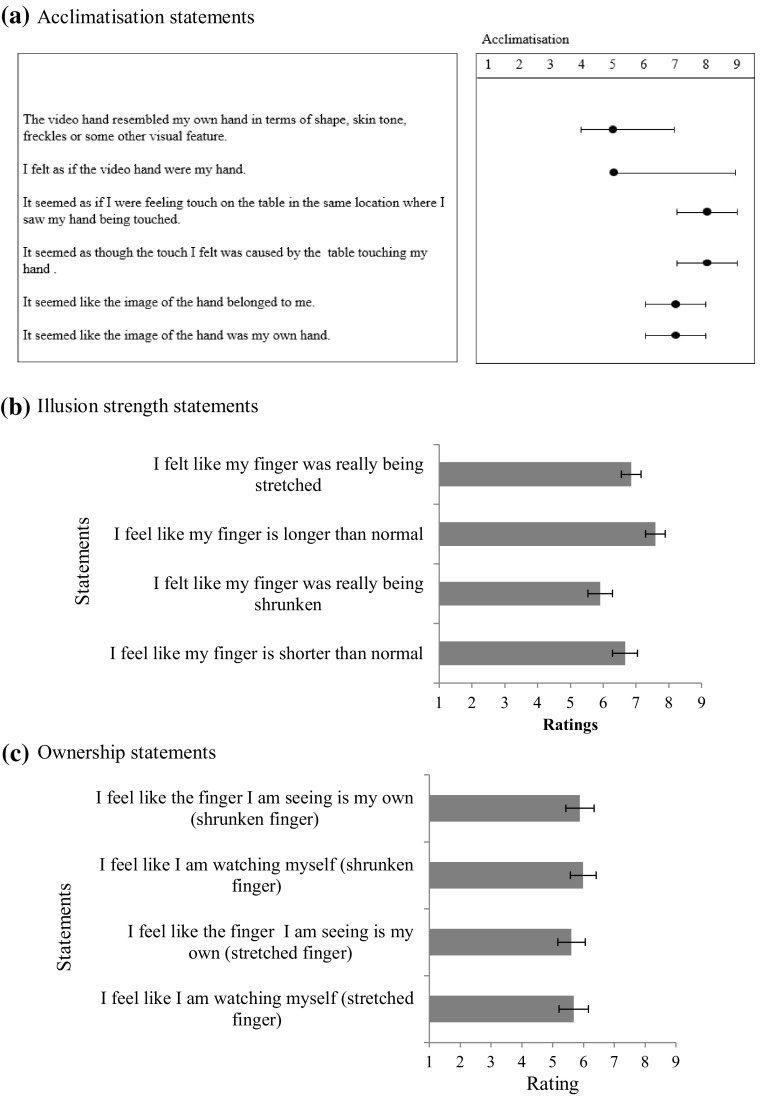
Mean questionnaire ratings: **a** medians and inter-quartile ranges for questionnaire ratings for acclimatisation. **b** Mean illusion strength and **c** ownership ratings for the stretched finger and shrunken finger illusions. *Error bars* represent standard error of the mean

As in Experiment 1, acclimatisation scores were not normally distributed (Shapiro Wilk statistic: *p* < 0.05) and remained so following attempts to transform the data; medians are, therefore, reported. Responses indicated a strong ownership towards the live video images of the hands (see Fig. 7a). Participants strongly agreed with statements such as ‘It seemed like the image of the hand was my own’ (median = 8) and ‘It seemed like the image of the hand belonged to me’ (median = 7).

##### Illusion questionnaires

Ratings indicated that participants felt their finger to be stretched or shrunk but still claimed ownership over these manipulated representations of the finger (see Fig. 7b, c). As in experiment 1, responses to the statements ‘I felt like my finger were being stretched/shrunken’ and ‘I feel like my finger is longer/shorter than normal’ were separately averaged for the stretched and shrunken finger conditions to obtain mean ratings for illusion strength. Ratings to the statements ‘I feel like I am watching myself’ and ‘I feel like the finger I am seeing belongs to me’ were separately averaged for both illusion conditions to obtain mean ratings of ownership. During the stretched finger condition, 96% of participants had average ratings of 5 or above for illusion strength, while 83% of participants had ratings of 5 and above during the shrunken finger condition. Mean ownership ratings indicated that 70 and 74% of participants had ratings of 5 or higher during the stretched and shrunken finger conditions, respectively. Less than 15% of the sample had scores of 3 or less in all conditions. Most participants, therefore, reported feeling each illusion and retained strong ownership over distorted representations of their finger. Although no difference between the illusion conditions was seen for mean ownership ratings (*t*_(22)_ = 0.81, *p* = 0.43; *d* = 0.14) comparing mean illusion strength statements revealed that stretching was felt more strongly than shrinking (*t*_(22)_ = 4.20, *p* < 0.001; *d* = 0.74).

#### Judgments of perceived finger length: online resizing

**Fig. 8:**
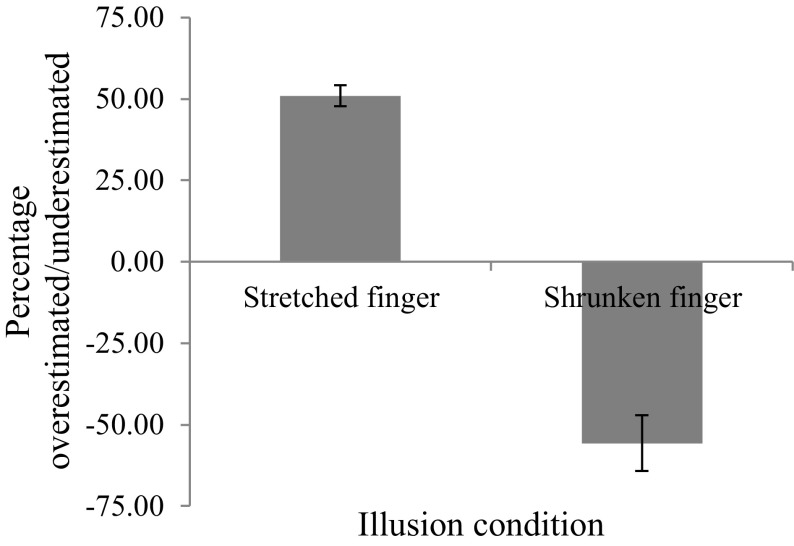
Percentage overestimation/underestimation of finger length following illusory manipulations (*error bars* show standard error of the mean)

Percentage increase or decrease in finger length from veridical was calculated and used to determine the mean percentage overestimation/underestimation for each participant in both conditions (see Fig. 8). As in Experiment 1, overestimation and underestimation were compared across both illusion conditions. Data remained not normally distributed (Shapiro Wilk statistic: *p* < 0.05); hence, non-parametric analyses were conducted. A Wilcoxon signed ranks test revealed no significant differences between percentage overestimation and underestimation of the finger following the stretched and shrunken illusions (*Z* = −1.43, *p* = 0.15). Chi square analyses were used to compare the proportion of participants that overestimated or underestimated perceived finger length in each condition separately. Following the stretched illusion, 96% of participants (*χ* ^2^ (1, *N* = 23) = 19.17, *p* < 0.001) overestimated finger length (mean percentage overestimation = 45.17%, SD = 26.29) stating that their finger had reached its veridical length when it was still much longer than in reality. Similarly, 91% of participants (*χ* ^2^ (1, *N* = 23) = 15.70, *p* < 0.001) underestimated their finger length following the shrunken illusion (mean percentage underestimation = 54.64%, SD = 41.45). One sample *t* tests revealed that perceived length overestimated (*t*_(21)_ = 8.98, *p* < 0.001, *d* = 1.91) and underestimated (*t*_(21)_ = 10.87, *p* < 0.001, *d* = 2.32) was significantly greater than zero (veridical finger length).

#### Judgments of perceived finger length: offline size estimation

Chi square analyses were again used to determine the proportion of participants that overestimated perceived real finger size following the stretched and shrunken illusions compared to initial length. 61% of participants overestimated perceived finger length compared to perceived initial finger length during the stretched illusion; however, this was not found to be significant (*χ* ^2^ (1, *N* = 23) = 1.09, *p* = 0.30; mean percentage overestimation = 17.23%, SD = 13.63; mean length overestimated = 11.9 mm). During the shrunken illusion, however, 83% of participants underestimated perceived finger length (*χ* ^2^ (1, *N* = 23) = 9.78, *p* = 0.002; mean percentage underestimation = 84.23%, SD = 11.01; mean length underestimated = 13.9 mm). Next, perceived length overestimated and underestimated was compared to perceived initial length. Perceived length following shrinking was found to be significantly shorter than perceived initial length (*t*(22) = 4.46, *p* < 0.001, *d* = 0.64); however, no difference between initial perceived length and perceived length following the stretched illusion was seen (*t*(22) = 1.70, *p* = 0.104).

In addition, we examined the association between percentage overestimation and underestimation for both tasks. Online and offline tasks were not correlated for percentage overestimation (*r*_(23)_ = −0.048, *p* = 0.83) or percentage underestimation (*r*_(23)_ = 0.34, *p* = 0.11) in perceived finger length, suggesting that the two tasks were in fact independent. In line with Experiment 1, the association between judgements of veridical finger length and illusion strength statements was again analysed, and the two were not found to be significantly correlated (all *p* > 0.05) suggesting differences between subjective and objective measures.

### Experiment 2: Discussion

In Experiment 2, we investigated how somatic representations were altered following manipulations to perceived body size using both online and offline measures of perceived body size as well as subjective ratings of illusion susceptibility and body ownership. As in Experiment 1, during the online task, perceived finger size was influenced by the nature of the illusion with longer and shorter representations of the finger being judged as veridical length following illusory stretching and shrinking in over 90% of the sample. However, the offline size estimation task only altered perceived veridical body size following the shrunken illusion, suggesting differences between various methods of measurement—in this case online and offline measures of perceived body size. Indeed, no significant associations between online and offline measures were evident for overestimations and underestimations of perceived finger length. Although these findings may not provide definitive evidence, the findings are consistent with the idea that online and offline size estimation tasks assess different aspects of the body representation—i.e. current perceptions of the body that are updated through incoming sensory input and stored perceptions of the body representation, respectively. The decrease in perceived body size for the offline measure nevertheless provides evidence suggesting that stored body representations may also be distorted, the reasons for which are addressed in the general discussion.

The questionnaire items demonstrated that the stretched illusion was felt more strongly compared to the shrunken. However, ownership ratings towards both manipulated representations of the finger were strong and no significant differences were observed for sense of ownership between the two conditions, indicating that ownership was not lost as a result of the distorted appearances of the hand, thus extending results of Experiment 1. Here again, subjective illusion strength ratings were not found to be associated with objective judgments of perceived body size following the illusions, perhaps suggesting that different mechanisms may underlie the two percepts.

## General discussion

The malleability of the body representation has been highlighted previously through clinical and experimental research, which has demonstrated an asymmetric tendency to embody larger body representations but not smaller ones (Haggard and Jundi 2009; Pavani and Zampini 2007; de Vignemont et al. 2005). Therefore over two experiments, we closely examined ratings of ownership over longer and shorter representations of the body, veridical body size perception following exposure to altered representations of the body, and whether differences in somatic size perception may be apparent across different methods of measurement.

### Illusion susceptibility and ownership of size altered body representations

In the current experiments participants were made to feel that their hand and index finger were a different length compared to their veridical finger/hand length, using visuo-proprioceptive illusions. Both experiments indicated that ownership was not lost as a result of the illusory manipulations. Although the questionnaire items demonstrated differences in illusion strength ratings during the stretched and shrunken finger conditions in Experiment 2, these illusion strength ratings were above the mid-value of 5 suggesting that participants felt each illusory manipulation and were susceptible to the illusions. This may reflect the fact that it was not technically possible to use a symmetrical size manipulation, as shrinking too far caused distortions of the first proximal knuckle which destroyed the illusion; however, participants still showed significantly reduced online estimations of veridical finger length after experiencing the shrinking illusion.

Furthermore, *ownership* ratings towards both manipulated representations of the finger and hand were strong and no significant differences in ownership were observed indicating that participants *felt* the distorted representations of their hand to belong to them. Findings of these studies also contradict early fake/rubber hand illusion studies that have shown asymmetric tendencies of ownership towards only larger representations of the body (Pavani and Zampini 2007; Haggard and Jundi 2009) and suggest that ownership is readily claimed over dynamic representations of own body parts even when reduced in size, perhaps due to its increased ecological validity and realistic appearance inducing a greater sense of ownership and/or agency than in previous studies. Moreover, as opposed to mere synchronous visuo-tactile information present in RHI studies, the illusions employed in the current studies provided additional sensory cues whereby the finger/hand was pulled or pushed with *force*/*pressure* that was in the direction of the manipulation and as a result may have induced stronger but convincing misperceptions of perceived body size.

### Judgments of perceived finger length: online resizing

Judgments of perceived finger and hand length during the online resizing task indicated that perceived body image was strongly affected by the nature of the illusions with longer and shorter hands/fingers being judged as veridical (or real) length following visuo-proprioceptive stretching and shrinking, respectively. These findings extend recent research that has reported embodiment of shrunken hands and bodies to have a scaling effect on the immediate environment (Bruno and Bertamini 2010; Linkenauger et al. 2013; Banakou et al. 2013). The fact that perceived body representation was influenced by the nature of the illusion also suggests that the stretched and shrunken illusions may have altered the mental representation of the hand, and extend previous virtual reality studies by providing direct evidence for the spontaneous flexibility of the body representation (i.e. without the need for scaling techniques). Whilst one may argue that the updated veridical body size perceptions reflected judgements of *where* the finger/hand was in space, rather than actual finger length, perceived body size was influenced by the illusions during the offline task, following the shrunken condition at least, thus suggesting that judgments were in fact reflective of perceived finger length.

### Judgments of perceived finger length: offline size estimation

When asked to indicate the perceived length of the finger using the divider (in Experiment 2), the difference between perceived initial length (prior to the illusions) and perceived length following illusions was only significant for the shrunken condition. This finding highlights differences between both online and offline methods of measurement. While online measures provide estimates of the body representation in its current form and is updated based on incoming sensory information, offline measures provide estimates of the typical perception of the body representation and is, therefore, thought to be relatively stable (Carruthers 2008). Perceived underestimation of finger length may, therefore, indicate that responses were again affected by nature of the illusion, thus suggesting that the mental representation of the body part was updated following the multisensory illusions. The absence of significant differences between perceived initial length and perceived length following stretching may suggest that offline body representation measures might have been stronger following illusory stretching, compared to shrinking. This could be because the long-term cortical representation of the body that evolves through development contains information relating to the shape and size of the body until it reaches adult size (O’Shaughnessy 1995; Melzack et al. 1997). As offline measures represent *stored body representations*, it may have prevented any significant overestimations in size following illusory stretching. In line with these findings, previous studies have also reported differences in perceived body shape and size with respect to the method of measurement in healthy and clinical populations (Cash and Deagle 1997; Longo and Haggard 2010, 2012).

### Implications and conclusion

Responsiveness to somatic illusions has previously been found to reflect the nature of underlying bodily distortions in clinical conditions (Thakkar et al. 2011; Keizer et al. 2014). For example, anorexia nervosa, in which disturbances in body shape and size are key features, is characterised by flexible body representations as evidenced by greater susceptibility to the RHI. Furthermore, induction of the RHI has also been found to alter such disturbances, making body representations more in keeping with correct or veridical body size, and thus highlights the importance of bodily illusions in both clinical assessments as well as potential therapeutic interventions (Keizer et al. 2014).

In conclusion, following multisensory distortions applied to participants’ own body, the current studies found mental body representations to be rapidly and directly updated to reflect the nature of the distortion. Importantly, ownership was retained for both the stretched and shrunken representations of the hand and finger. Susceptibility to somatic illusions and the ability to retain ownership over altered perceptions of the body provide a potential mechanism for the treatment of a range of clinical conditions in which the identity and integrity of the body have been compromised.

## Footnotes

1 The divider was handed with the two points closed. During the offline body size estimates, participants were asked to estimate how long they felt their finger to ‘really’ be as opposed to what they were seeing.
